# Efficacy of Reuterin and Bacteriocins Nisin and Pediocin in the Preservation of Raw Milk from Dairy Farms

**DOI:** 10.17113/ftb.58.04.20.6728

**Published:** 2020-12

**Authors:** Nirmal Kumar, Vinay Kumar, Syed Mohsin Waheed, Diwas Pradhan

**Affiliations:** 1 Department of Biotechnology, Graphic Era (Deemed to be University), 566/6 Bell Road, Society Area, Clement Town, 248002 Dehradun, Uttarakhand, India; 2 Dairy Microbiology Division, ICAR-National Dairy Research Institute, GT Rd, 132001 Karnal, Haryana, India

**Keywords:** reuterin, bacteriocins, raw milk, milk biopreservation, *Lactobacillus reuteri*

## Abstract

**Research background:**

In the current scenario of milk production in developing and developed countries, several factors influence the shelf-life of raw milk and add significant numbers of microbial contaminants that drastically lower the initial microbial quality leading to milk spoilage by the time it reaches the processing units.

**Experimental approach:**

The present study was undertaken to investigate the biopreservative efficacy of reuterin system along with different combinations of bacteriocins in controlling the initial microflora of raw milk at farm level. *Lactobacillus reuteri* strain LR47, having effective antimicrobial activity, was shortlisted from our previous study and further characterized for reuterin production and tested in raw milk system.

**Results and conclusions:**

Preliminary testing of the cell-free supernatant from *L. reuteri* LR47 demonstrated significant growth inhibition of the majority of the tested bacterial indicators of milk spoilage. Further genetic analysis of the *L. reuteri* LR47 revealed the presence of two genes (*pduC* and *dhaB*) involved in the utilization of glycerol to produce reuterin *via* two different pathways. The strain LR47 was also found to possess comparatively higher capacity to convert glycerol into reuterin when checked through colorimetric assay. In the raw milk biopreservation experiment with reuterin alone or in combination with bacteriocins, the highest level of growth suppression in the total bacterial load and coliform counts was observed in the sample that was treated with a combination of reuterin, nisin and pediocin. The treatment combining these three natural biopreservatives at specific concentrations was able to maintain the initial microbial quality and extend the shelf-life of raw milk by 6 h at 37 °C based on the microbial counts and physicochemical properties, *viz*. pH and titratable acidity. In conclusion, the results confirm that the use of reuterin in combination with bacteriocins is a promising approach for temporary control of the raw milk microflora and extension of its shelf-life until further processing.

**Novelty and scientific contribution:**

This study demonstrates for the first time the use of reuterin for the extension of shelf-life of raw milk as an alternative treatment method.

## INTRODUCTION

Milk is an important component of the diet of a vast population. It ranks high among other foods and is considered as the perfect food for humans from birth to old age. It not only has good sensory properties, but it also fulfils the nutrient requirements of the body for healthy growth. It could also prevent or reduce risk of many nutritional deficiency diseases (*1*). Milk, due to its unique composition and growth-promoting properties, is a favourable growth medium for many microorganisms responsible for milk-borne diseases and its spoilage (*2*), and for the same reason, it is highly perishable item. Poor handling of milk may affect public health and cause economic loss, thus it requires hygienic vigilance and monitoring throughout the chain (*3*). Milk from healthy cows is virtually sterile before leaving the udder (*4*). Number and type of bacteria that might contaminate milk immediately after milking depend on the cleanliness and hygienic practices of the farmworkers, hygiene of milking equipment and dairy farm environment, weaknesses in the cold chain during production process, transportation, storage, *etc.* (*5*).

The presence of high microbial load in the raw milk is a serious problem because of the ability of bacteria to cause milk-borne diseases and reduce the shelf-life of milk. Furthermore, contaminated milk is also responsible for spreading milk-borne epidemics to humans. According to World Health Organization (WHO) reports, raw milk is implicated in 39.1% of the total bacterial foodborne outbreaks (*6*). In fact, over the past few decades, the number of reported foodborne disease outbreaks have significantly increased, which is attributed to raw milk (*7*). Microbial contamination of milk is responsible for economic losses at various stages of the milk production chain. For instance, early spoilage of milk in developing countries was responsible for the loss of 20% of milk production, resulting in a 12 US$ per month loss to each dairy farmer (*8*). According to the Food and Agriculture Organization (FAO) report, 90 million US$ worth of milk is lost each year in East Africa and Near East during production and distribution before they even reach a store (*9*). As per the 2010 US Department of Agriculture data, 17 billion pounds of milk, or 32% of total supply, is lost in a year because of milk spoilage (*10*). Moreover, a study carried out in the North West of Cameroon showed that each farmer loses 1 L of milk every day because of high microbial contamination of raw milk (*8*). To overcome this economic distress, both developing and developed countries are experimenting with different approaches to control the microbial load to prevent loss of raw milk at the farm level. Natural antimicrobial compounds are an attractive, safe and cost-effective alternative to prevent milk wastage. However, information available on the biopreservation of raw milk using natural antimicrobial compounds is very limited.

Metabolites of lactic acid bacteria (LAB) are used to improve the safety and extend the shelf-life of different dairy products. Interest in the LAB metabolites has increased exponentially in the last few years. Many different bacteriocins and their combinations with other antimicrobial compounds have been tried to inactivate milk-borne pathogens or spoilage microorganisms with the aim to preserve milk and its products (*11*). Bacteriocins produced by LAB have antibacterial activity and contain generally recognized as safe (GRAS) compounds (*12*). Thus, they are increasingly used as natural food preservatives. Nisin and pediocin are bacteriocins that are now available commercially under the name Nisaplin^TM^ (*13*) and Alta 2341^TM^ (*14*), respectively. However, most bacteriocins have rather small spectrum of activity, being active mainly against Gram-positive bacteria (*15*). Hence, many researchers have attempted to incorporate bacteriocins along with other treatments to broaden their activity, using the concept of hurdle technology for controlling spoilage and pathogenic microorganisms in many food items. Some studies have shown synergistic activity of bacteriocins (*16*) and their combinations with other antimicrobial agents such as reuterin (*17*). Despite the strong antimicrobial activity of many LAB metabolites, no sufficient studies have been conducted to maintain the microbial quality of raw milk, particularly at dairy farm level.

*Lactobacillus reuteri* found in the gastrointestinal (GI) tract of humans and many other animals is one of the important species of lactobacilli (*18*). *L. reuteri* is known to be a natural component of human milk (*19*) and is also found in fermented foods (*20*). The strains of *L. reuteri* possess a unique property of anaerobically converting glycerol into a potent, broad-spectrum antimicrobial substance, termed 3-hydroxypropionaldehyde (3-HPA), which is commonly known as reuterin (*21*). Reuterin is an aldehyde that exhibits a wide range of antimicrobial activity against foodborne pathogens and spoilage microorganisms (*22*). It has a high potential as a food biopreservative since it is water-soluble, resists degradation by proteolytic and lipolytic enzymes, and withstands a broad range of pH and temperature conditions (*23*). The antimicrobial efficacy of reuterin has been demonstrated against many common foodborne spoilage and pathogenic organisms such as *Staphylococcus aureus, Escherichia coli, Listeria monocytogenes, Salmonella* Typhi, *Enterococcus feacalis* and *Pseudomonas aeruginosa* (*24*). Reuterin has a wider range of antimicrobial activity than bacteriocins and other non-bacteriocin antimicrobial compounds (*25*). Reuterin and nisin in combination act synergistically against *L. monocytogenes* and additively against *S. aureus* in milk (*26*). Reuterin, alone or in combination with nisin and lactoperoxidase system (LPS), exhibited inhibitory activity against *E. coli* O157:H7, *S. enterica*, *C. jejuni*, *A. hydrophila* and *Y. enterocolitica* in refrigerated milk. The combination of monolaurin and nisin has been found to be active against bacilli in milk; in particular, the inhibition of *B. licheniformis* by both antimicrobial substances increased with increasing pH when they were added simultaneously to milk (*27*). In addition, the combination of both compounds successfully exerted a bactericidal effect against different *Bacillus* species in skimmed milk, and also inhibited their regrowth and sporulation (*28*). The combination of LPS and nisin had a synergistic and long-lasting inhibitory effect on *L. monocytogenes* in reconstituted skimmed milk and, also, its effectiveness did not depend on pH (*29*). Therefore, the incorporation of reuterin and its combination with other bacteriocins in milk and milk products offers a superior alternative for improving the safety, quality, and shelf-life of milk along with the elimination of chemical preservatives. Addition of antimicrobial compounds to raw milk may help in resolving shelf-life problems caused by inadequate refrigeration/absence of cooling facilities, long-distance transport under hot weather conditions *etc*. (*23*). Thus, the present work investigates the preventive role of reuterin in raw milk spoilage under tropical conditions where temperatures above 30 °C are normally prevalent during summers. Therefore, all experiments were conducted at 37 °C. Until now, no such study has been conducted on the preservation of raw milk using wide-spectrum antimicrobial compounds and their combination with other bacteriocins. Hence, the current study aims to explore the possibility of applying an antimicrobial mix with reuterin as the main additive to extend the shelf-life of raw milk and reduce the probability of milk-borne pathogenesis.

## MATERIALS AND METHODS

### Bacterial strains and culture conditions

*Lactobacillus reuteri* ATCC 55730 obtained from BioGaia, Stockholm, Sweden, was used as a reference strain and the test strain, *L. reuteri* LR47 was selected from our previous study (*30*). The bacterial indicator strains (obtained from an in-house lab culture collection) *Escherichia coli* K12, *Listeria monocytogenes* ATCC 13932, *Enterococcus faecalis* NCDC 135, *Serratia marcescens* ATCC 13880, *Salmonella* Typhi NCDC 113, *Yersinia enterocolitica* ATCC 130715, *Klebsiella pneumoniae* NCDC 138 and *Pediococcus acidilactici* LB42 were used for the antibacterial activity testing of *L. reuteri* LR47 supernatant. All the bacterial cultures (excluding *P. acidilactici* LB42) were grown in brain heart infusion broth (BHI, HiMedia, Mumbai, India), while *P. acidilactici* LB42 and *L. reuteri* strains were grown in de Man, Rogosa and Sharpe broth (MRS; HiMedia, Mumbai, India) at 37 °C for 24-48 h. The bacterial strains were maintained as 15% glycerol stock cultures at -20 °C in BHI and MRS broths. The organisms were propagated twice before their use in the experiments.

### Preparation of cell-free supernatants of L. reuteri LR47

Cell-free supernatants (CFS) of *L. reuteri* LR47 were prepared according to the method described by Lüthi-Peng *et al.* (*31*). A single colony with typical morphology (small pinpointed and creamy white colony) from the MRS agar plates was picked and inoculated into MRS broth and incubated anaerobically (gas pack system, HiMedia) at 37 °C for 20 h. After incubation, the culture was centrifuged (Precision Biotech Instruments Pvt. Ltd., Delhi, India) at 4000×*g* and 4 °C for 10 min. The supernatant was filter-sterilised through a 0.22-μm syringe filter (Millipore, New Delhi, India) to remove any remaining cells. The cell pellet was used for secondary fermentation to prepare a suspension of MRS supplemented with glycerol (360 mM). The supernatant obtained from secondary fermentation of MRS with glycerol was designated MRS-Gly, while the supernatant derived from MRS broth was designated MRSB. Finally, the pH values of the above supernatants were determined using a portable pH meter (Orion Star™ A111; Thermo Fisher Scientific, Waltham, MA, USA). The pH of the supernatants was adjusted from pH=4.6 to 6.5 (in order to eliminate the cause of microbial death due to acidic pH) using 0.1 M sodium hydroxide at room temperature prior to testing their antimicrobial activity.

### Antibacterial activity of L. reuteri LR47 supernatant

Antibacterial activity of *L. reuteri* LR47 CFS was previously tested in our study (*30*) using agar well diffusion method (*32*). In this study, we used the microtiter plate method to test the antibacterial activity of the CFS. An aliquot of 20 μL of CFS was added, in triplicate, into 180 μL of BHI broth containing 10^6^ CFU/mL of each pathogen, and incubated at 37 °C for 18 h. Negative control contained pathogen cultures only, while positive control contained commercial antibiotics (*γ*(ampicillin)=100 mg/mL as stock solution; Sigma-Aldrich, St. Louis, MO, USA) instead of CFS. The inhibition of indicator strains was quantitatively determined by measuring the absorbance (*A*) (Spectrostar Nano BMG Labtech, Ortenberg, Germany) at 620 nm, in comparison with the control. The percentage of inhibition was calculated as follows:

FTB-58-359-e1.eps

### PCR detection of reuterin-producing pduC and dhaB genes in L. reuteri LR47

PCR was conducted using genomic DNA of *L. reuteri* LR47 to detect the presence of *dhaB* (part of *dha* gene operon) and *pduC* (gene coding for large fragment of *gdh* protein) genes, responsible for conversion of glycerol into reuterin, using DHAB1 (5’-AACTACGATAACATGTTTGC-‘3), DHAB7 (5’-CCTTCTTCTTCAATTCCGGCA-‘3) and pduCF (5’-CCTGAAGTAAAYCGCATCTT-‘3), pduCR (5’-GAAACYATTTCAGTTTATGG-‘3) primer pair as per the method described by Versalovic *et al*. (*33*) and Walter (*34*), respectively. The resulting PCR products were separated and visualized using standard agarose gel electrophoresis.

### Colourimetric assay for reuterin and glycerol quantification

CFSs (MRS-Gly) of three *L. reuteri* strains (*L. reuteri* LR47, *L. reuteri* LR11 and *L. reuteri* LR 49) were assayed to quantitatively determine glycerol utilisation and the resulting concentration of reuterin present in the CFS. Reuterin and glycerol concentrations were determined, in triplicate, using a colourimetric method adapted from Spagnola (*35*). Briefly, an aliquot of 330 µL of CFS (MRS-Gly) from each bacterium was mixed with 225 µL of 10 mM tryptophan (dissolved in 0.05 M HCl) and 150 µL of 95% ethanol. Each mixture was incubated at 40 °C for 50 min and the absorbance was measured at *A*_560 nm_ using spectrophotometer microplate reader (Spectrostar Nano BMG Labtech, Ortenberg, Germany). The reuterin concentration was calculated by comparing the absorbance of the samples with a standard curve of acrolein, assuming that 1 mol of reuterin was dehydrated to 1 mol of acrolein that reacted with tryptophan in the presence of HCl.

### Determination of L. reuteri LR47 cell-free supernatant activity

The activity of *L. reuteri* LR47 CFS was quantified by minimum inhibitory concentration (MIC) assay using a microtiter plate against *E. coli* K12 as described by Chung *et al.* (*36*). Overnight culture of *E. coli* K12 was harvested, washed twice with phosphate buffer (pH=7.2, 50 mM), suspended in the same buffer, and diluted to an *A*_620 nm_ of 0.2 measured using a spectrophotometer (Spectrostar Nano BMG Labtech). This suspension was diluted 1:10, which corresponds to about 10^6^ CFU/mL. The diluted suspension (0.1 mL) was used to inoculate 0.2 mL of serial dilutions of CFS in Müller-Hinton medium (HiMedia). The microtiter plate was incubated at 37 °C for 12 h and then the growth of indicator strain *E. coli* K12 was examined. The unit of the CFS activity was defined as the reciprocal of the highest dilution that did not permit visible growth of the indicator strain and expressed in arbitrary unit (AU). Accordingly, the activity of the CFS was calculated to be 1600 AU/mL.

### Measurement of pediocin and nisin activities

Pediocin was prepared by inoculating active cultures of pediocin-producing *Pediococcus pentosaceus* 34 (*37*) in 100-mL aliquots of MRS broth and incubated at 37 °C for 24 h. CFS was prepared by centrifugation of the culture in a refrigerated centrifuge (Precision Biotech Instruments Pvt. Ltd.) at 4000×*g* for 10 min. The supernatant was filter-sterilized by passing through 0.22-µm pore size membrane filter after neutralization. Pediocin activity was quantified by the MIC method against *Pediococcus acidilactici* LB42 as described by Kaur *et al*. (*38*) using microtiter plates. Nisin was procured from Sigma-Aldrich. Stock solutions of nisin (1000 IU/mL) were prepared in 0.02 M HCl and stored at -40 °C. The nisin stock solution was diluted prior to use in experiments.

### Collection of raw cow’s milk samples and experimental design

Raw milk samples of *Bos taurus* (Sahiwal breed) were obtained from the Experimental Dairy plant of ICAR-NDRI, Karnal, India. All samples were collected in the morning. After the collection of samples, it was immediately transported to the laboratory in a tightly closed container placed in an ice box. At the start of each experiment, a volume of 100 mL of raw milk was aliquoted in 14 sterile screw-cap glass bottles as per experimental design. Each sample was marked with the type of antimicrobial treatment to be given. Antimicrobial compounds in different combinations and different activities (Table 1) were added concomitantly to the respective sample bottles. Milk sample without the addition of antimicrobials served as a control. All the untreated and treated milk samples were incubated at 37 °C for 9 h and parameters were measured after 3, 6 and 9 h of experiment.

**Table 1:** Activity of reuterin (R) in combination with nisin (N) and pediocin (P)

Treatment	Reuterin activity/(AU/mL)	Nisin activity/(IU/mL)	Pediocin activity/(AU/mL)
Control	0	0	0
R1	150	0	0
N1	0	100	0
P1	0	0	2185
R1+N1	150	100	0
R1+P1	100	0	2185
R1+N1+P1	150	100	2185
R2+N2+P2	16	20	600
R1+N2+P2	150	20	600
R2+N1+P1	16	100	2185
R1+N1+P2	150	100	600
R2+N2+P1	16	20	2185
R2+N1+P2	16	100	600
R1+N2+P1	100	20	2185

### Chemical and bacteriological analyses

The analysed chemical parameters of the raw milk samples were pH and titratable acidity (TA). The pH was measured using a digital pH meter (Orion Star™ A111; Thermo Fisher Scientific) and the TA was determined by titration method (*39*) at different time intervals (0, 3, 6 and 9 h). Each sample was measured in triplicate. Total plate count (TPC) and coliform counts were also tested simultaneously by the sample dilution pour plate method. Briefly, tenfold serial dilutions of samples were made up to volume fraction of 10^-7^ in normal saline solutions (0.85%). A volume of 1 mL of milk sample was diluted in a series of normal saline solutions (9 mL of saline down to 10^-8^) and 1-mL aliquots of milk in the saline solution from appropriate volume fraction dilutions: 10^-1^, 10^-3^ or  10^-5^ for coliform count, and 10^-3^, 10^-5^ or 10^-7^ for TPC were transferred to Petri dishes. Samples were plated in triplicates, using pour plate technique. Nutrient agar medium (HiMedia) was used for the TPC, while violet red bile agar (HiMedia) was used for counting coliforms incubated at 37 °C. After incubation, plates containing 30 to 300 colonies were selected for screening and results were expressed as log CFU/mL.

### Statistical analysis

The data obtained in this study are presented as mean value±standard deviation (S.D.) and analyzed statistically with the GraphPad Prism software (*40*). Microbiological counts were converted to log CFU/mL and the statistical significance among different antimicrobials and their combination in milk system was compared by two-way analysis of variance (ANOVA) followed by Tukey’s test and considered significant at p≤0.01.

## RESULTS AND DISCUSSION

### Antibacterial activity of L. reuteri LR47 CFS against selected indicator strains

**Fig. 1:**
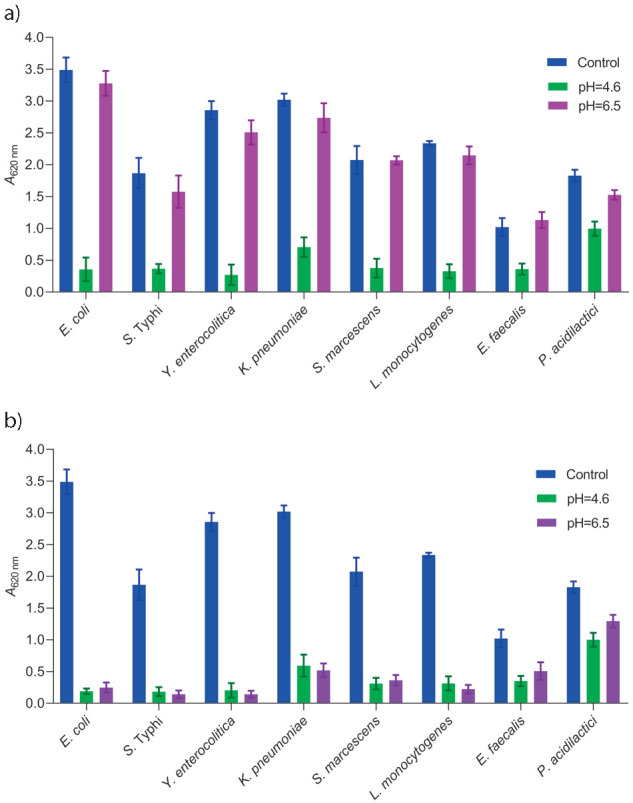
Indicator strains cocultured with *Lactobacillus reuteri* LR47 cell-free supernatant (both MRSB and MRS-Gly) and tested for inhibitory effects. The growth of each pathogen was determined by measuring *A*_620 nm_ after 18 h of incubation at 37 °C: a) inhibitory effect of MRSB with (pH=6.5) and without (pH=4.6) pH adjustment, and b) inhibitory effect of MRS-Gly with and without pH adjustment. Untreated controls contained pathogen cultures only. Bar values are presented as calculated mean value of measurements at *A*_620 nm_ (*N*=3) with standard error of the mean shown as vertical bars

Many wild-type variants of *Lactobacillus reuteri* have the ability to produce reuterin from anaerobic conversion of glycerol (*22*). Reuterin is a potent and a wide-spectrum antimicrobial agent that suppresses various kinds of bacteria, fungi, protozoa, *etc.* (*21*). However, many inherent genetic factors as well as other external factors influence reuterin production (*41*). Hence, efforts are mainly focused on bioprospect high reuterin-producing *L. reuteri* strains from different niches. We selected *L. reuteri* strain LR47 from our previous study (*30*), which had shown highest antimicrobial activity during the initial screening and antimicrobial activity, which was further tested against eight different bacterial indicators that included important food pathogens. Both, CFS before (pH=4.5) and after the adjustment of the pH value (pH=6.5) derived from MRSB and MRS-Gly were used to test the antimicrobial activity. Antimicrobial activity was observed in both MRS-Gly and MRSB (Fig. 1); however, inhibitory effect of MRSB is very low or insignificant at pH=6.5 (Fig. 1a). *L. reuteri,* being a LAB, secretes organic acids such as lactic acid, acetic acid, *etc.* as primary fermentation products and many others as secondary metabolites (*15*) that have been reported to produce strong antimicrobial agents, particularly in a relatively low pH environment, which may have contributed to the antimicrobial activity observed in the MRSB. However, antimicrobial activity could not be replicated at pH=6.5. On the other hand, CFS (pH_adjusted_=6.5) from MRS-Gly exhibited strong antimicrobial activity against the majority of indicator strains. It was able to strongly inhibit both Gram-negative and Gram-positive bacterial indicators. The activity observed in this case can be attributed to reuterin, since it is used as an antimicrobial agent against a wide range of bacterial targets at different pH values including neutral pH (*42*), contrary to organic acids and bacteriocins that are effective only against a limited number of bacterial targets and often lose their potential at an altered pH. However, reuterin did not have any significant inhibitory effect against *E. faecalis* and *P. acidilactici*. This is not surprising as it has been reported that reuterin affects lactic acid bacteria to a lesser degree, and a relatively higher concentration of reuterin is required to inhibit the growth of LAB (*21*).

### PCR detection of reuterin-producing genes in L. reuteri LR47

Few bacterial strains have the metabolic capacity to utilize glycerol and produce reuterin. Among them are *L. reuteri* strains, which possess all the necessary genes for bioconversion of glycerol into reuterin. Glycerol dehydratase and diol dehydratase are two discrete isofunctional enzymes, which catalyse the reaction of glycerol dehydration to reuterin by two independent pathways (*41*, *43*). The structural genes of the glycerol dehydratase (*dhaBCE*) of *L. reuteri* are part of the *dha* regulon and its expression is induced under anaerobic conditions in the presence of glycerol. On the other hand, diol dehydratase genes of *L. reuteri* are part of *pdu* operon (*pduCDE*) which also contains genes for glycerol utilization. Glycerol, a second substrate of *pduCDE*, is transformed into an intermediate compound (reuterin), which can be further metabolized to 1,3-propanediol or 3-hydroxypropionate. Earlier, Schaefer *et al*. (*44*) demonstrated the elimination of reuterin production by disrupting/deleting the *pduC* gene in *L. reuteri* strains ATCC 23272 and confirming the role of *pduC* gene in reuterin production. Therefore, we also attempted to check the presence of either one or both genes in *L. reuteri* LR47 by PCR assay. The PCR results showed a positive amplification of both genes, *viz. pduC* (105 bp) and *dha* (900 bp) in *L. reuteri* LR47 (data not shown), which confirmed that this strain possessed these genes and molecular set-up to drive the conversion of glycerol to reuterin.

### Colourimetric estimation of reuterin production in L. reuteri strains

**Fig. 2:**
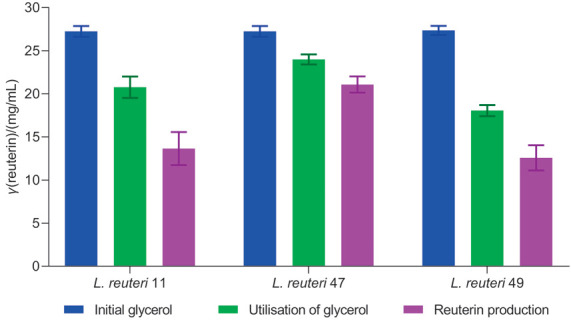
Comparison of reuterin production among three selected *Lactobacillus reuteri* strains. Reuterin was produced by *L. reuteri* strains LR11, LR47 and LR49 in glycerol solution using a two-step fermentation process. Reuterin concentrations were determined using a modified colourimetric method and compared against the concentrations of glycerol remaining in the solution to yield a percentage of glycerol converted to reuterin. The blue bars represent initial glycerol concentration, green bars represent utilisation of glycerol during reuterin production, and the purple bars indicate the reuterin production form the utilised glycerol. *L. reuteri* 47 strain indicates that the amount of produced reuterin is significantly different (p<0.05) than the amounts produced by other strains

To confirm and augment the genetic screening results, we also performed the colourimetric estimation of reuterin production in CFS of *L. reuteri* LR47. Colourimetric estimation of reuterin was performed to determine the levels of reuterin produced among different strains of *L. reuteri* (*L. reuteri* LR47, *L. reuteri* LR49 and *L. reuteri* LR11) (*30*). Initial and final concentrations of reuterin were determined and compared within and among different strains used in the study. Also, the amount of utilised glycerol was determined and compared with the respective supernatant of these three *L. reuteri* strains. It is evident from Fig. 2 that reuterin production in *L. reuteri* LR47 was significantly higher than in the other two *L. reuteri* strains. The different levels of reuterin production (12.58, 13.65 and 21.08 mg/mL) could also correlate well with the differences in the glycerol utilization (18.06 20.77 and 24.00 mg/mL) of *L. reuteri* LR49, LR11 and LR47, respectively. Other research groups have reported reuterin production in the range of 45.7 to 298 mM (*30*, *41*, *42*). However, most of these studies have used different *L. reuteri* strains and conditions, and also *E. coli* system to stimulate reuterin production. In contrast, we carried out a direct assay in MRS-Gly broth for reuterin production and obtained quite significant reuterin concentrations without any external stimulation. This process can be particularly useful in *in situ* biopreservation of food where antimicrobial production is required. Nevertheless, the *E. coli* system could also be tested here to achieve a still higher reuterin concentrations in *L. reuteri* LR47 strain.

### Effect of reuterin and bacteriocin treatments on raw milk shelf-life

**Fig. 3:**
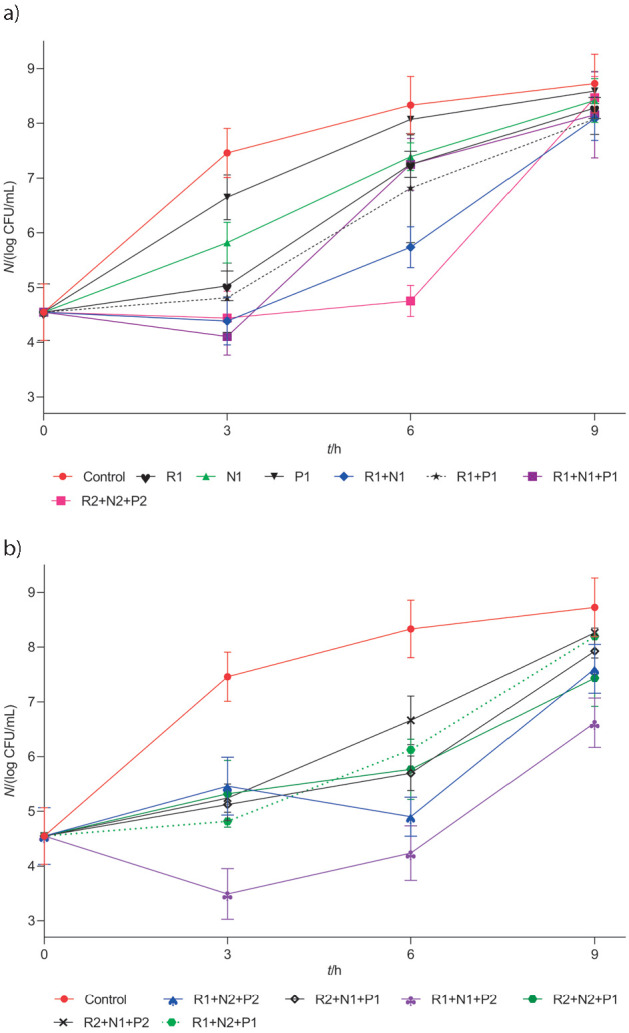
Inhibitory activity of different biopreservative treatments on total plate count (TPC) (log CFU/mL) of raw milk at different time intervals at 37 °C: a) when treated with first and b) second set of biopreservatives (reuterin (R), nisin (N) and pediocin (P)). Results are shown as mean values±standard deviations (S.D.) of three samples taken from three replicate experiments (*N*=9). Sample abbreviations are given in Table 1

**Fig. 4:**
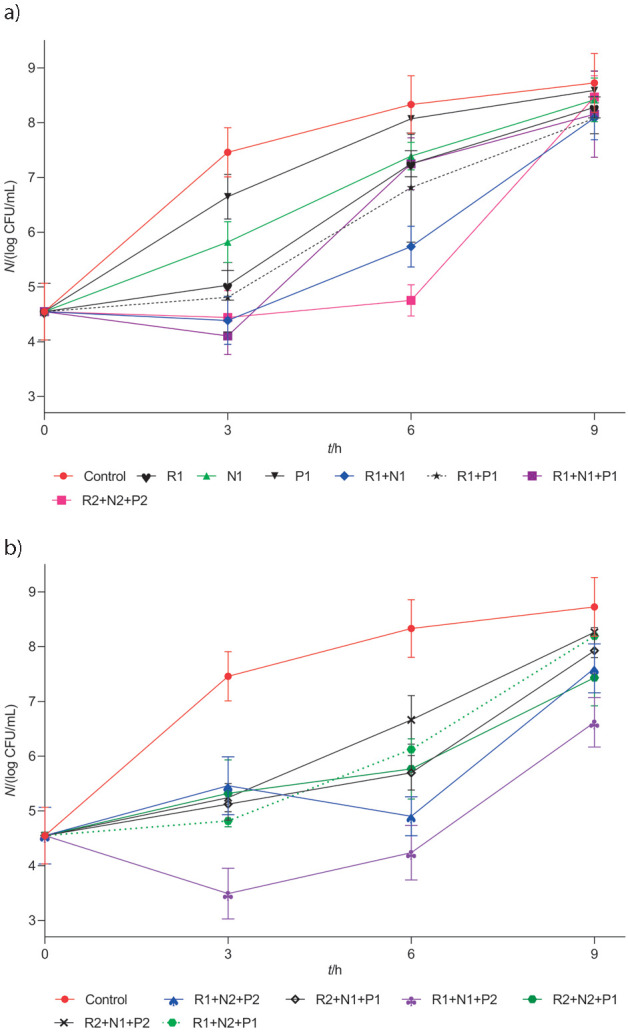
Inhibitory activity of different biopreservative treatments on coliform count (log CFU/mL) of raw milk at different time intervals at 37 °C: a) when treated with first and b) second set of biopreservatives (reuterin (R), nisin (N) and pediocin (P)). Results are shown as mean values±standard deviations (S.D.) of three samples taken from three replicate experiments (*N*=9). Sample abbreviations are given in Table 1

Based on the literature survey on reuterin and bacteriocins (*11*-*13*, *17*, *24*, *26*, *29*), current work was undertaken to investigate the effect of different concentrations of reuterin, nisin and pediocin individually or in different combinations on the shelf-life of raw milk. Literature search indicates that there are hardly any studies conducted on the preservation of raw milk using reuterin and bacteriocins. Thus, we proceeded to experiment and verify if different combinations of these antimicrobial compounds would be effective in controlling the initial microbial counts without compromising the physiochemical properties of raw milk. The selected concentrations of reuterin, nisin and pediocin were based on the earlier studies (*11*, *17*, *29*) and total plate count (TPC), coliform count, pH and titratable acidity (TA) were measured to monitor microbial progression at 0, 3, 6 and 9 h of incubation at 37 °C. Raw milk samples were significantly influenced by the type of antimicrobial combination in the treatments and the duration of incubation at 37 °C (shown in Fig. 3 and Fig. 4, Table S1 and Table S2).

In the first subgroup, reuterin, nisin and pediocin were added singly to each raw milk sample and physicochemical parameters were measured. At the beginning of the incubation period (0 h), the overall TPC and coliform count was 4.6 and 4.1 log CFU/mL, respectively, in all raw milk samples. However, after the initial 3-hour incubation, there was a significant increase in the TPC and coliform count in the control sample. The addition of reuterin, nisin and pediocin showed an initial bacteriostatic effect, having respective TPC and coliform count of 2.4, 1.6 and 0.7 log CFU/mL and 1.1, 1.5 and 0.4 log CFU/mL (Fig. 3a and Fig. 4a), which was lower than control sample count (Table S1 and Table S2). After further 3-hour incubation (*i.e*. total 6 h of incubation), the TPC and coliform count continued to increase in the control sample, as well as in the treated samples, but they remained lower than in the control. After 9 h of incubation, the antibacterial action of the three added biopreservatives seemed to have been exhausted and very low bacterial inhibition was visible in the treated samples. Earlier, EI-Ziney and Debevere (*45*) and Arqués *et al.* (*24*), who studied the efficacy of reuterin in UHT skimmed milk at 7 or 37 °C, had shown that reuterin had a bacteriostatic effect against individual Gram-negative bacteria. However, it was also observed that reuterin enacted strong inhibitory action against other pathogens and inhibition of up to 4 log units could be attained, but at a lower temperature of 7 °C. Arqués *et al.* (*11*) in another study showed that when added singly, nisin exhibited bactericidal effect against *S. aureus* at 37 °C during 4 h of incubation. However, the pathogen regained its growth after reaching bacterial counts similar to that in control milk after 24 h. Hence, it is quite clear that individual antimicrobial metabolites have limited capacity against bacterial targets in the milk system. Inhibitory effect can be more suppressed when mixed microflora in ambient conditions are used, like in this study. Hence, the different combinations of reuterin, nisin and pediocin were further tested to achieve better results.

In the second subgroup, a combination of two antimicrobial compounds was used. A greater initial inhibition of TPC and coliform count was observed than when these antimicrobial compounds were applied individually in raw milk system. After 3 h, the raw milk treated with 150 AU/mL reuterin and 100 IU/mL nisin (R1+N1), and 150 AU/mL reuterin and 2185 AU/mL pediocin (R1+P1) had TPC and coliform counts of 3.2 and 2.2 log CFU/mL and 2.6, 1.5 log CFU/mL respectively, which was lower than the control sample (Fig. 3a and Fig. 4a). However, at the end of 6 h of incubation, the initial inhibitory effect of the combination started diminishing and there was a steady increase in the TPC and coliform count in the samples treated with both combinations. Thereafter, the growth resumed in the two samples after 9 h of incubation and the TPC and coliform bacteria reached counts that were comparable with those in the control sample. The initial reduction in the bacterial counts was synergistic because the combined effect of two treatments was greater than the sum of individual inhibition treatments. However, some researchers have reported that the combined effect of reuterin with nisin could not improve the antimicrobial efficacy of reuterin against Gram-negative bacteria in UHT skimmed milk at 4 or 8 °C, although they also reported that reuterin in combination with nisin had a synergistic effect against *L. monocytogenes* in milk at 37 °C (*25*). Arqués *et al.* (*26*) also reported a synergistic effect of reuterin (8 AU/mL) with nisin (100 IU/mL) against *L. monocytogenes* and *S. aureus* in milk at 37 °C. The reuterin activity against *L. monocytogenes* has also been reported to be enhanced by the addition of 3% salt (*45*). Hence, the results of the current study confirm the synergistic action of reuterin and nisin against the raw milk microflora. In addition, a combination of nisin and pediocin PA-1 also showed a strong synergistic bactericidal activity against milk pathogens (*46*). However, the dual combination was not effective enough to obtain a sufficient level of shelf-life extension of raw milk at 37 °C, hence, triple combinations were further tested.

In the third subgroup with the combination of three biopreservatives at different concentrations, the treatment with 150 AU/mL reuterin, 100 IU/mL nisin and 600 AU/mL pediocin (R1+N1+P2) showed the best results and had a significant effect on the TPC and coliform count. After 3 h of incubation, the TPC and coliform count were 3.9 and 2.9 log CFU/mL respectively, which is lower than in the control milk (Fig. 3b and Fig. 4b). The efficacy of 150 AU/mL reuterin, 100 IU/mL nisin and 600 AU/mL pediocin (R1+N1+P2) treatment was maintained even after 6 h of incubation and very minimal increase in the TPC and coliform count was observed. The effect, however, seemed to fade thereafter and increase in both TPC and coliform count could be observed (Fig. 3b and Fig. 4b). As anticipated, the triple combination of antimicrobial compounds showed the best effect, which could be attributed to a synergistic action among the used antimicrobials. Arqués *et al.* (*11*) had shown earlier that a similar triple combination consisting of three biopreservatives reuterin, nisin and lactoperoxidase system (LPS) in curdled milk had a very strong synergistic bactericidal effect against *L. monocytogenes* with only 0.3 log CFU/mL bacterial count remaining in the milk samples compared to control (8.3 log CFU/mL). In compliance with our obtained results, they also reported that the combination of three biopreservatives comprising both reuterin and nisin along with LPS system exhibited superior action to single or a combination of two biopreservatives in milk system.

**Fig. 5:**
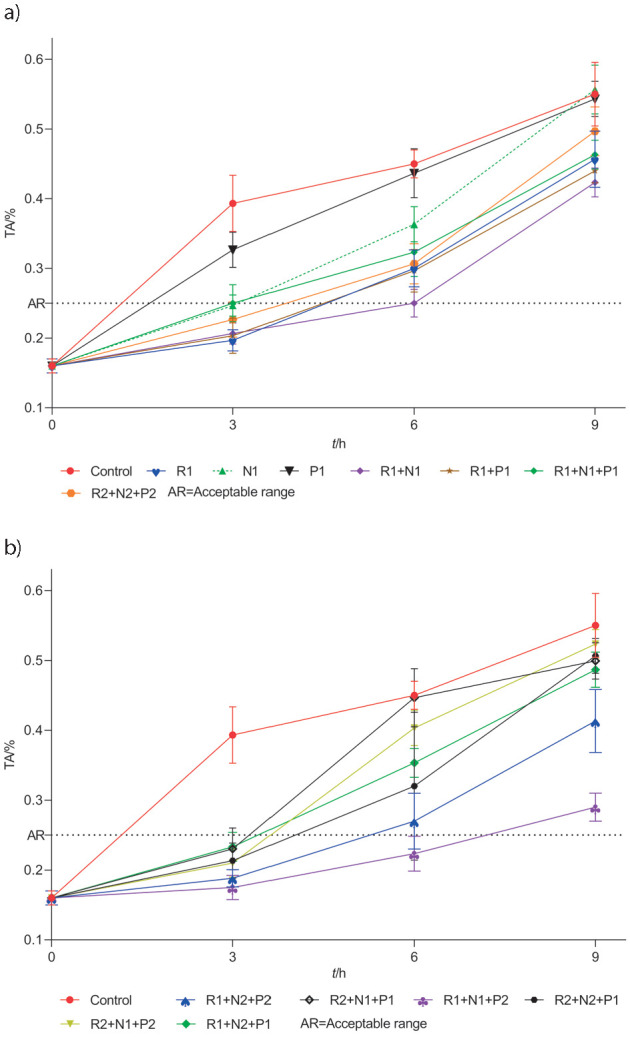
Change in titratable acidity (TA) of raw milk after different biopreservative (reuterin (R), nisin (N) and pediocin (P)) treatments at different time intervals at 37 °C: a) with first and b) second set of biopreservatives. Results are shown as mean values±standard deviations (S.D.) of three samples taken from three replicate experiments (*N*=9). Sample abbreviations are given in Table 1

**Fig. 6:**
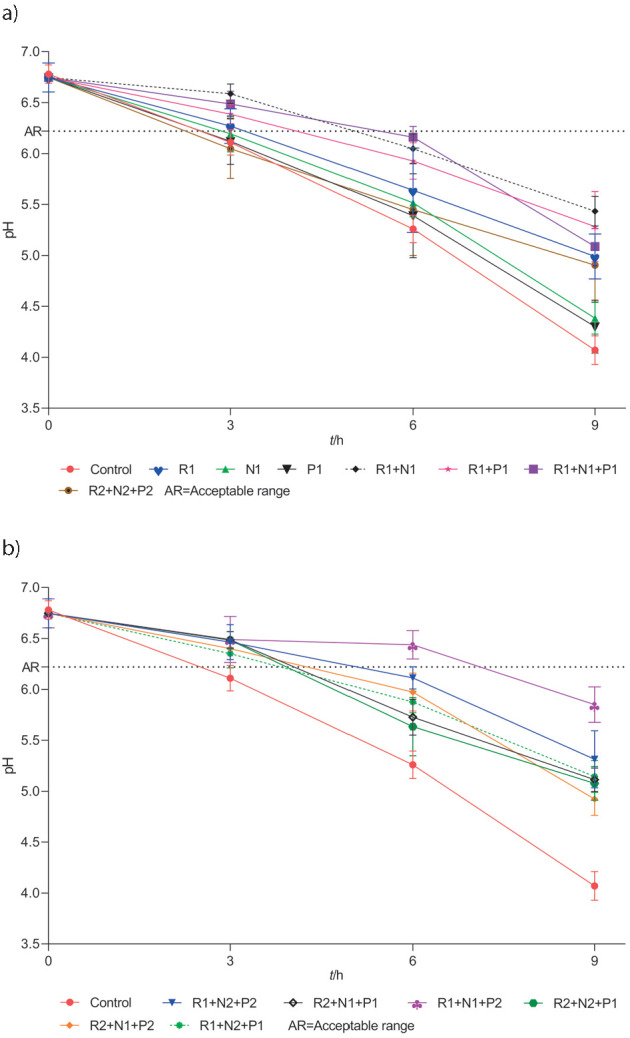
Change in the pH of raw milk after different biopreservative (reuterin (R), nisin (N) and pediocin (P)) treatments at different time intervals at 37 °C: a) when treated with first and b) second set of biopreservatives. Results are shown as mean values±standard deviations (S.D.) of three samples taken from three replicate experiments (*N*=9). Sample abbreviations are given in Table 1

Efficacy of biopreservatives used in triple combinations is evident in the pH and acidity of the treated raw milk samples. Among different combinations of biopreservatives, only the treatment with R1+N1+P2 combination was able to maintain TA of 0.22% lactic acid (Fig. 5) and pH=6.43 (Fig. 6) after 6 h of incubation, which was within the commercially acceptable range of pH=6.22-6.77 and TA of 0.16-0.25% lactic acid (*47*), while after all the other treatments higher pH and TA values exceeded their commercially acceptable limits (Fig. 5 and Fig. 6). Under the prevailing tropical conditions, the major bacterial spoilage encountered in raw milk is curdling, which is mainly due to the increase in titratable acidity and decrease of pH. The commercial dairy industry also uses TA values to judge the freshness of raw milk as a basis for its overall acceptability for further processing. The rise in TA values is a direct indicator of microbiological activity in milk, which is more rapid at ambient temperatures (*48*). Apart from various other microbial groups, the LAB groups and coliforms are the most active in the initial increase in the TA of raw milk. Bacteriocins used in the study such as nisin and pediocin are derived from LAB groups and are active against closely related species (*15*). Hence, bacteriocins used in the study may have played an important role in controlling the LAB groups. However, bacteriocins are not very effective against Gram-negative bacteria such as coliforms, *Pseudomonas* and other similar bacteria that are highly prevalent in raw milk. Hence, the reuterin system may have worked in parallel to inhibit and suppress the growth of these groups of bacteria in the raw milk and extend the shelf-life of raw milk for a period of 6 h at 37 °C (Table S3 and Table S4). The reuterin system works by inducing oxidative stress in target cells (*44*), while both bacteriocins typically act by forming pores in the bacterial cell membrane. This multitarget system could also explain the increased efficacy and synergistic action of the triple combination of biopreservatives in raw milk system. Thus, our results show that reuterin could be successfully combined with nisin and pediocin at specific concentrations and used to inhibit complex microflora that is prevalent in raw milk and maintain it for at least 6 h under ambient temperature conditions.

## CONCLUSIONS

Current study was initiated to prevent spoilage of raw milk as heavy microbial contamination of raw milk at the farm level is the major reason of milk spoilage under the prevailing tropical conditions. In the absence of suitable technological interventions, the use of natural biopreservatives, *viz.* lactic acid bacteria (LAB) and their metabolites, can offer a suitable solution to address this problem. During the course of the study, *Lactobacillus reuteri* strain LR47 was characterized for its antimicrobial activity, presence of reuterin-producing genes, and its capability to produce reuterin, a potent wide-spectrum antimicrobial metabolite, was confirmed. Finally, the ability of reuterin-containing cell-free supernatants from *L. reuteri* LR47 in conjunction with bacteriocins to enhance the shelf-life of raw milk under ambient conditions was tested. The results show that a combination of specific concentrations of reuterin, nisin and pediocin could effectively control the initial microbial load in raw milk and prolong the shelf-life up to 6 h at 37 °C, while maintaining the physicochemical parameters, *viz*. acidity and pH, within the commercially acceptable range. The study is the first of its kind to demonstrate that wide-spectrum antimicrobial metabolite(s) from LAB have a great potential to improve and maintain the quality and shelf-life of raw milk at farm level and minimize economic losses to milk farmers and dairy industry sector. Further studies may be required to optimize the use of reuterin for extending the shelf-life of raw milk beyond 6 h and establish its safety and efficacy as a biopreservative. If successful, this approach could be a first line of milk preservation at farm level even before undergoing any thermal/radiation or extended shelf-life processing or entering the cold chain.

## Figures and Tables

**Table S1:** Change in the total plate count (log CFU/mL) of raw milk after different biopreservative (reuterin (R), nisin (N) and pediocin (P)) treatments at different time intervals at 37 °C

Treatment	0	*t*/h 3 *N*/(log CFU/mL)	6	9
C	(4.6±0.5)^aA^	(7.5±0.4)^aB^	(8.3±0.5)^aB^	(8.9±0.5)^ADC^
R1	(4.6±0.5)^aA^	(5.0±0.3)^bA^	(7.3±0.2)^aB^	(8.2±0.2)^AC^
N1	(4.6±0.5)^aA^	(5.9±0.4)^baB^	(7.4±0.3)^aC^	(8.5±0.4)^AD^
P1	(4.6±0.5)^aA^	(6.8±0.4)^baB^	(8.1±0.3)^aC^	(8.7±0.4)^ADC^
R1+N1	(4.6±0.5)^aA^	(4.3±0.4)^bcA^	(5.9±0.4)^bB^	(8.4±0.4)^AC^
R1+P1	(4.6±0.5)^aA^	(4.9±0.6)^baA^	(6.5±0.9)^aB^	(8.3±0.3)^AC^
R1+N1+P1	(4.6±0.5)^aA^	(4.1±0.3)^bdA^	(4.9±0.5)^aC^	(7.2±0.8)^ADC^
R2+N2+P2	(4.6±0.5)^aA^	(4.4±0.5)^beA^	(4.8±0.3)^cA^	(8.0±0.4)^AB^
R1+N2+P2	(4.6±0.5)^aA^	(5.3±0.5)^baA^	(4.9±0.4)^dA^	(7.7±0.3)^AB^
R2+N1+P1	(4.6±0.5)^aA^	(5.0±0.5)^bfA^	(5.7±0.4)^eBA^	(7.9±0.5)^AC^
R1+N1+P2	(4.6±0.5)^aA^	(3.6±0.5)^bgB^	(4.2±0.5)^fAB^	(6.7±0.5)^AC^
R2+N2+P1	(4.6±0.5)^aA^	(5.2±0.6)^baA^	(5.9±0.4)^aBA^	(7.3±0.5)^AC^
R2+N1+P2	(4.6±0.5)^aA^	(5.2±0.3)^bhA^	(6.8±0.4)^aB^	(8.1±0.5)^AC^
R1+N2+P1	(4.6±0.5)^aA^	(4.9±0.6)^baA^	(6.1±0.3)^aB^	(8.2±0.3)^AC^

Results are shown as mean values±standard deviations (S.D.) of three samples taken from three replicate experiments (*N*=9). Mean values within the same row with different superscripted uppercase letters differ significantly (p<0.01) among treatments for a given time. Mean values within the same column with different superscripted lowercase letters differ significantly (p<0.01) among treatments for a given time. Activity/(AU/mL): R1=150, R2=16, P1=2185 and P2=600; Activity/(IU/mL): N1=100 and N2=20

**Table S2:** Change in coliform count (log CFU/mL) of raw milk after different biopreservative (reuterin (R), nisin (N) and pediocin (P)) treatments at different time intervals at 37 °C

Treatment	0	*t*/h 3 *N*/(log CFU/mL)	6	9
C	(4.1±0.1)^aA^	(6.4±0.4)^aB^	(7.8±0.4)^aC^	(8.2±0.5)^aDC^
R1	(4.1±0.1)^aA^	(5.2±0.5)^bA^	(6.1±0.2)^bBA^	(8.0±0.4)^aAB^
N1	(4.1±0.1)^aA^	(4.9±0.7)^baA^	(6.2±0.4)^aB^	(7.5±0.6)^aC^
P1	(4.1±0.1)^aA^	(5.9±0.5)^bcB^	(6.9±0.8)^aAB^	(8.2±0.4)^aCBA^
R1+N1	(4.1±0.1)^aA^	(4.1±0.6)^bdAC^	(5.3±0.5)^cACD^	(7.1±0.6)^aBCD^
R1+P1	(4.1±0.3)^aA^	(4.9±0.5)^ebAC^	(6.9±0.7)^aAD^	(7.1±0.5)^aBCD^
R1+N1+P1	(4.1±0.2)^aA^	(4.3±0.6)^bfA^	(4.9±0.7)^dAB^	(6.9±0.7)^bAC^
R2+N2+P2	(4.1±0.2)^aA^	(4.9±0.9)^gbAD^	(5.8±0.5)^eBD^	(6.6±0.5)^cC^
R1+N2+P2	(4.1±0.1)^aA^	(3.8±0.5)^bhAC^	(4.8±0.6)^fAD^	(6.9±0.5)^dBCE^
R2+N1+P1	(4.1±0.1)^aA^	(4.9±0.6)^biAD^	(5.0±0.4)^gB^	(6.6±0.5)^eCDB^
R1+N1+P2	(4.1±0.1)^aA^	(3.5±0.5)^bjAC^	(3.9±0.4)^hAD^	(6.3±0.4)^fB^
R2+N2+P1	(4.1±0.1)^aA^	(4.9±0.6)^bkA^	(4.3±0.4)^iA^	(6.8±0.9)^gA^
R2+N1+P2	(4.1±0.1)^aA^	(4.4±0.5)^blA^	(5.5±0.5)^jAC^	(7.0±0.4)^hBC^
R1+N2+P1	(4.1±0.1)^aA^	(3.9±0.6)^bmA^	(6.2±0.5)^kAC^	(6.8±0.5)^iBC^

Results are shown as mean values±standard deviations (S.D.) of three samples taken from three replicate experiments (*N*=9). Mean values within the same column with different superscripted lowercase letters differ significantly (p<0.01) among treatments for a given time. Mean values within the same row with different superscripted uppercase letters differ significantly (p<0.01) among treatments for a given time. Activity/(AU/mL): R1=150, R2=16, P1=2185 and P2=600; Activity/(IU/mL): N1=100 and N2=20

**Table S3:** Change in pH of raw milk after different biopreservative (reuterin (R), nisin (N) and pediocin (P)) treatments at different time intervals at 37 °C

Treatment	0	*t*/h 3	6	9
C	(6.7±0.1)^aA^	(6.1±0.1)^aB^	(5.3±0.1)^aC^	(4.1±0.1)^aD^
R1	(6.7±0.1)^aA^	(6.3±0.2)^aB^	(5.6±0.4)^aC^	(5.0±0.2)^aD^
N1	(6.7±0.1)^aA^	(6.2±0.2)^aB^	(5.5±0.1)^aC^	(4.4±0.2)^aD^
P1	(6.7±0.1)^aA^	(6.1±0.2)^aB^	(5.4±0.4)^bC^	(4.3±0.3)^bD^
R1+N1	(6.7±0.1)^aA^	(6.6±0.1)^bA^	(6.0±0.2)^cB^	(5.4±0.2)^aC^
R1+P1	(6.7±0.1)^aA^	(6.4±0.1)^aA^	(5.9±0.2)^dB^	(5.3±0.4)^cC^
R1+N1+P1	(6.7±0.1)^aA^	(6.5±0.1)^aAB^	(6.2±0.1)^aB^	(5.1±0.2)^aC^
R2+N2+P2	(6.7±0.1)^aA^	(6.0±0.3)^aB^	(5.5±0.5)^eC^	(4.9±0.4)^dD^
R1+N2+P2	(6.7±0.1)^aA^	(6.5±0.2)^aAB^	(6.1±0.1)^aB^	(5.3±0.3)^eC^
R2+N1+P1	(6.7±0.1)^aA^	(6.5±0.1)^aA^	(5.7±0.2)^fB^	(5.1±0.1)^fC^
R1+N1+P2	(6.7±0.1)^aA^	(6.5±0.2)^aA^	(6.4±0.1)^aA^	(5.9±0.2)^gB^
R2+N2+P1	(6.7±0.1)^aA^	(6.4±0.1)^aA^	(5.6±0.3)^gB^	(5.1±0.2)^hC^
R2+N1+P2	(6.7±0.1)^aA^	(6.4±0.1)^aA^	(6.0±0.2)^hB^	(4.9±0.2)^iB^
R1+N2+P1	(6.7±0.1)^aA^	(6.4±0.2)^aA^	(5.9±0.1)^iB^	(5.1±0.2)^jB^

Results are shown as mean values±standard deviations (S.D.) of three samples taken from three replicate experiments (*N*=9). Mean values within the same column with different superscripted lowercase letters differ significantly (p<0.01) among treatments for a given time. Mean values within the same row with different superscripts uppercase letter differ significantly (p<0.01) among treatments for a given time. Activity/(AU/mL): R1=150, R2=16, P1=2185 and P2=600; Activity/(IU/mL): N1=100 and N2=20

**Table S4:** Change in titratable acidity (TA) of raw milk after different biopreservative (reuterin (R), nisin (N) and pediocin (P)) treatments at different time intervals at 37 °C

Treatment	0	*t*/h 3 TA/%	6	9
C	(0.16±0.01)^aA^	(0.39±0.04)^aB^	(0.45±0.02)^aCB^	(0.55±0.046)^aDC^
R1	(0.16±0.01)^aA^	(0.20±0.02)^aB^	(0.30±0.03)^aC^	(0.46±0.04)^aD^
N1	(0.16±0.01)^aA^	(0.25±0.02)^aB^	(0.36±0.03)^aC^	(0.56±0.04)^aD^
P1	(0.16±0.01)^aA^	(0.33±0.03)^bB^	(0.44±0.04)^bCB^	(0.54±0.03)^aD^
R1+N1	(0.16±0.01)^aA^	(0.21±0.02)^cB^	(0.25±0.02)^cAB^	(0.42±0.02)^aC^
R1+P1	(0.16±0.01)^aA^	(0.20±0.03)^dA^	(0.30±0.03)^dB^	(0.44±0.02)^aC^
R1+N1+P1	(0.16±0.01)^aA^	(0.25±0.03)^eB^	(0.32±0.04)^eAB^	(0.46±0.02)^aC^
R2+N2+P2	(0.16±0.01)^aA^	(0.23±0.03)^fB^	(0.31±0.03)^fC^	(0.50±0.04)^aD^
R1+N2+P2	(0.16±0.01)^aA^	(0.19±0.01)^gB^	(0.27±0.04)^gAB^	(0.41±0.05)^aC^
R2+N1+P1	(0.16±0.01)^aA^	(0.23±0.03)^hA^	(0.45±0.04)^aB^	(0.50±0.03)^aCB^
R1+N1+P2	(0.16±0.01)^aA^	(0.18±0.02)^iA^	(0.22±0.03)^hB^	(0.29±0.02)^bC^
R2+N2+P1	(0.16±0.01)^aA^	(0.21±0.03)^jA^	(0.32±0.11)^aAB^	(0.51±0.03)^aB^
R2+N1+P2	(0.16±0.01)^aA^	(0.21±0.02)^kB^	(0.40±0.03)^aC^	(0.52±0.02)^aD^
R1+N2+P1	(0.16±0.01)^aA^	(0.23±0.02)^lB^	(0.35±0.02)^iC^	(0.49±0.03)^aD^

Results are shown as mean values±standard deviations (S.D.) of three samples taken from three replicate experiments (*N*=9). Mean values within the same column with different superscripted lowercase letters differ significantly (p<0.01) among treatments for a given time (Dennett’s test). Mean values within the same row with different superscripted uppercase letters differ significantly (p<0.01) among treatments for a given time. Activity/(AU/mL): R1=150, R2=16, P1=2185 and P2=600; Activity/(IU/mL): N1=100 and N2=20
